# Yield survey and nutritional evaluation of garlic stalk for ruminant feed

**DOI:** 10.1186/s40781-017-0147-3

**Published:** 2017-10-23

**Authors:** Y. H. Lee, Y. I. Kim, Y. K. Oh, F. Ahmadi, W. S. Kwak

**Affiliations:** 1 Division of Food Bio-science, College of Medical Life Sciences, Konkuk University, Chungju, Chung-Buk, Republic of Korea; 2 Animal Nutrition & Physiology Team, National Institute of Animal Science, Rural Development Administration, Wanju county, 565–851 Jeon-Buk province Republic of Korea

**Keywords:** Garlic stalk, Nutritional value, Rice straw, Ruminal degradation, Yield

## Abstract

**Background:**

Very limited information exists on the ruminal degradation kinetics of nutrients in garlic stalk. The present study aimed to survey the annual yield of garlic stalk in Korea and determine its feed-nutritive value for ruminants.

**Methods:**

In Experiment 1, garlic stalk was incubated in situ in the rumen of two Hanwoo steers (360 ± 15 kg body weight) and removed after 12, 24, or 48 h to determine the ruminal degradation kinetics of DM and NDF. Rice straw was also included for comparison. In Experiment 2, In Experiment 2, six male Corriedale sheep were randomized to two dietary treatments to determine the apparent digestibility of nutrients in garlic stalk. Diets included a control ration without garlic stalk (60% concentrate mix +40% ryegrass) or a treatment ration (70% control diet +30% garlic stalk).

**Results:**

The Korean national yield of garlic stalk (sun-dried basis) in 2016 was estimated to be 31,910 tons, with the southern coastal regions producing the highest quantity. Compared with rice straw, garlic stalk had lower NDF, higher ADF, and greater effective degradabilities of DM and NDF, resulting in a greater TDN value (56.3%), which was higher than that obtained for rice straw (43.7%).

**Conclusion:**

These results provide basic information on the ruminal DM and NDF degradation kinetics of garlic stalk, which would be helpful for the efficient utilization of this by-product in ruminant diets.

## Background

Garlic (*Allium sativum* L.) is a flavoring spice with an annual production of approximately 20 million tons in the world, with China, India, and Korea being the main producers [1]. A recent review [2] on garlic processing waste reported the increasing trend in global demand for garlic consumption, leading to the substantial waste production [25–30% solid waste], where husk and straw (collectively known as garlic stalk) constitute the major waste by-product. However, garlic stalk, generated abundantly during the harvesting period, is usually made into compost or incinerated [3, 4]. Therefore, problems associated with disposal costs and environmental pollution are emerging, and solutions are needed [5].

To efficiently utilize garlic processing by-product and mitigate the environmental concerns associated with its accumulation in landfills, garlic by-products have been evaluated as a biomass for bioenergy production [6], soil amendment [7] or bio-adsorbent [8]. However, the use of garlic by-products as a source of animal feed is still the most promising and viable route for efficient utilization of this waste resource [2]. Previous studies on garlic stalk silage have indicated that it contains a moderate amount of protein (9.3–13.0%) and a high concentration of NDF (48–59%) [4, 9, 10], which may be a potential source of roughage for ruminant animals. In general, the use of human-inedible resources, such as garlic stalk, as a source of animal feed would help to minimize the problem of feed inadequacy, potentially reducing feed costs, which constitute the majority of production costs, and reducing the problems associated with the disposal of this waste resource. However, to efficiently utilize garlic stalk in ruminant diets, accurate information on the ruminal degradation kinetics of its nutrients is required. Recently, Kamruzzaman et al. [10] reported that replacing 10% of hay (orchardgrass and reed canarygrass) with garlic stem and leaf silage in sheep diet resulted in greater N and energy utilization without detrimental effects on ruminal fermentation. Chu et al. [9] reported an improvement in meat quality and economic income when garlic stalk silage was fed to steers during the fattening period. More recently, Panthee et al. [11] reported that inclusion of garlic leaves at 2.5 g/(kg BW^0.75^·d) had no negative effects on ruminal fermentation characteristics and had positive N utilization in sheep. These studies investigated the feed value of garlic stalk silage for beef cattle, or the bioactive components in garlic stem and leaf silage for sheep. However, to our knowledge, no information exists on the ruminal degradation kinetics of nutrients in garlic stalk. Therefore, the current study was undertaken to estimate the yield of garlic stalk in Korea, and to evaluate ruminal degradation kinetics and the feeding value of garlic stalk for sheep.

## Methods

### Annual yield estimation

The annual garlic yield in the major garlic-cultivating provinces in Korea was surveyed in four consecutive years (2013–2016) to estimate the quantity of garlic stalk production. Survey data were obtained from the National Statistics Office [12]. The yield of garlic stalk (wet basis) was estimated as a proportion of total garlic production [total garlic production × 73.6%]. The annual yield of garlic stalk (sun-dried basis) was calculated according to the following equation:

$$ \mathrm{Yield}\  \mathrm{of}\ \mathrm{sun}\hbox{-} \mathrm{dried}\  \mathrm{garlic}\  \mathrm{stalk}=\frac{\mathrm{Yield}\  \mathrm{of}\ \mathrm{wet}\ \mathrm{garlic}\  \mathrm{stalk}\times \mathrm{DM}\ \mathrm{content}\ \left(\%\right)\ \mathrm{of}\ \mathrm{wet}\ \mathrm{garlic}\  \mathrm{stalk}}{\mathrm{DM}\ \mathrm{content}\ \left(\%\right)\ \mathrm{of}\  \mathrm{garlic}\  \mathrm{stalk}\ \left(\mathrm{sun}\hbox{-} \mathrm{dried}\  \mathrm{basis}\right)} $$

The DM content of wet and sun-dried garlic stalk was 13.6 and 86.4%, respectively.

### Sample collection and chemical analyses

Garlic stalk (sun-dried) was obtained from a garlic processing plant located in Uysung County, Kyungbuk Province, and an agricultural fishery marketing center located in Chungju city (Chungbuk Province, Korea). To ensure representative sampling, the samples were collected eight times between July 2015 and February 2016. Prior to chemical analysis, samples were dried and then ground to pass through a 1-mm screen (Cemotec, Tecator, Skanor, Sweden). The DM, CP, ether extract (EE), crude fiber (CF), ash, NDF (with α-amylase and sodium sulfite), and ADF contents were determined according to the standard methods of the Association of Official Analytical Chemists [13]. Lignin was measured after 72% (*w*/w) sulfuric acid solubilization of cellulose. Nitrogen associated with ADF (acid detergent insoluble CP; ADICP) was measured according to the methods described by Licitra et al. [14]. True protein was measured following the precipitation of N fractions in trichloroacetic acid solution (5%).

### Experiment 1: In situ study

All animal care protocols were approved by the Konkuk University Institutional Animal Care and Use Committee. Two cannulated Hanwoo steers (body weight = 360 ± 15 kg; mean ± standard deviation) were used for the in situ experiment, which was conducted in three separate runs on different days. The animals were fed 5 kg of a corn-based concentrate mix (DM 89.8%, CP 14.3%, EE 3.0%, crude ash 7.5%, CF 6.26%, NDF 29.6%, and ADF 17.2%) and 2 kg of rice straw daily to meet the nutrient requirements for early fattening steers [15]. The in situ trial was performed according to the method of Ørskov et al. [16] as previously described in detail [17]. In brief, the experimental samples were milled to pass through a 2-mm sieve (Cemotec, Tecator, Skanor, Sweden), and then a 5-g sample (DM basis) was placed into a Dacron bag (10 × 20 cm, 53 ± 10-μm pore size; R1020, Ankom Technology, Macedon, NY, USA). Two-hours after the morning feeding, the bags were incubated in triplicate, in the ventral ruminal sac for 12, 24, or 48 h. At the end of the incubation, bags were retrieved, washed under running cold water until the water leaving the bag was clear, and then dried in an oven at 55 °C for 48 h until analysis. The soluble A fraction (washout fraction) was determined after the 0-h bags were washed in a washing machine (25 °C for 40 min). The in situ DM and NDF data were fitted to the following first-order kinetic model [16]:

$$ \mathrm{Y}=\mathrm{A}+\mathrm{B}\left[1\hbox{-} {\mathrm{e}}^{\hbox{-} K\mathrm{d}(t)}\right] $$

where, Y = ruminal disappearance rate at time *t*; A = immediately soluble fraction; B = potentially degradable fraction; *K*_d_ = degradation rate of the degradable B fraction; and *t* = incubation time.

### Experiment 2: In vivo study

Six Corriedale rams (body weight = 51.0 ± 2.0 kg; mean ± standard deviation) were distributed randomly to receive one of two diets (14-day adaptation period and 7-day collection period). Dietary treatments included a control diet (60% concentrate mix +40% ryegrass; 11.2% CP, 1.9% EE, 22.8% CF, 55.6% NDF, 35.7% ADF, 7.6% crude ash, and 56.5% NFE) and a treatment diet, where 30% of the control diet was replaced with garlic stalk (70% control diet +30% garlic stalk; 9.7% CP, 1.8% EE, 26.7% CF, 54.0% NDF, 39.9% ADF, 10.1% crude ash, and 51.8% NFE). The chemical compositions of the concentrate mix, ryegrass, and garlic stalk, used in the formulation of experimental diets, are listed in Table 1. Diets were mixed daily and offered (1.7% of body weight; 850 g DM basis) twice daily in equal portions at 07:00 and 18:00 h. Diets were formulated to meet the nutrient requirement of sheep [18]. Animals always had free access to fresh water. During the experiment, the animals were kept in individual metabolism crates (1.6 × 0.5 m), which permitted the collection of feces. Daily fecal output during the collection period was dried at 65 °C for 48 h. Feces were thoroughly mixed at the end of the collection period to obtain a composite sample. The TDN value in garlic stalk was estimated using the values of digestible CP, EE, CF, and NFE [19].

**Table 1:** Chemical composition of ingredients fed to sheep

Item, % of DM	Concentrate mix	Ryegrass straw	Garlic stalk
Dry matter, %	87.5	91.4	88.8
Organic matter	90.6	95.0	84.0
Ether extract	2.91	0.53	1.40
Crude protein (CP)	15.6	4.52	6.21
True protein, % of CP	87.4	87.2	79.8
NPN^a^, % of CP	12.6	12.8	20.2
ADICP^b^, % of CP	24.7	43.4	37.3
Neutral detergent fiber	39.1	80.4	50.2
Acid detergent fiber	23.4	54.3	49.6
Hemicellulose	15.7	26.0	0.63
Crude fiber	10.4	41.3	35.7
Ash	9.40	5.02	16.0
Nitrogen-free extract	61.7	48.7	40.7

^a^ *NPN* Non-protein nitrogen

^b^ *ADICP* Acid detergent insoluble CP

### Statistical analysis

Experimental feeds were sampled eight times during 2015–2016, with the following resultant experimental design: two feeds (garlic stalk and rice straw) × eight batches × three analytical replicates (per feed per batch), giving a total of 48 observations. The data were analyzed according to the following model: Y_ij_ = μ + F_i_ + R_j_ + e_ij_, where, Y_ij_ = response variable; μ = mean; F_i_ = fixed effect of feeds (*i* = 2); R_j_ = random effect of batch (j = 8); and e_ij_ = error term.

In situ data were obtained over the course of three runs in different days, resulting in the following experimental design: three incubation runs × two feeds (garlic stalk and rice straw) × two animal replicates × three sample replicates, giving a total of 36 observations. The data were analyzed according to the following model: Y_ij_ = μ + F_i_ + R_j_ + e_ij_, where, Y_ij_ = response variable; μ = mean; F_i_ = fixed effect of feeds (*i* = 2); R_j_ = random effect of incubation run (j = 3); and e_ij_ = error term. When a significant difference was found, least-squares means were separated using Student’s *t*-test [20]. A *P*-value less than 0.05 was considered significant.

## Results

### Annual yield of sun-dried garlic stalk

The annual yield of garlic stalk production (from 2013 to 2016) in major provinces in Korea is shown in Table 2. An approximate quantity of 303,297 tons of garlic stalk (wet basis) was produced in 2013, which then followed a decreasing trend until 2015. An estimated quantity of 31,910 tons of garlic stalk (sun-dried) was generated in 2016, when the highest amount was produced in Gyeongnam, accounting for 25.4% of the total production quantity, followed by Jeonnam, Gyeongbuk, Chungnam, and Jeju.

**Table 2:** Production quantity of garlic stalk in Korean provinces: 2013–2016

Item, tons	Year			
	2013	2014	2015	2016
Production of garlic stalk (wet basis)				
Metropolis^a^	4352	3957	2983	6484
Gyeonggi	5448	4657	4454	4536
Gangwon	2604	1877	1755	1068
Chungbuk	4150	3200	3149	3596
Chungnam	29,671	25,729	20,190	23,065
Jeonbuk	5285	6357	5046	7688
Jeonnam	80,386	70,925	48,600	43,621
Gyeongbuk	50,208	43,997	33,843	40,354
Gyeongnam	80,259	66,401	52,979	51,407
Jeju	40,934	33,167	22,902	20,904
Total production of garlic stalk^b^ (wet basis)	303,297	260,267	195,900	202,723
Total production of garlic stalk^c^ (sun-dried basis)	47,741	40,968	30,836	31,910

^a^Seoul, Busan, Daegu, Incheon, Gwangju, Daejeon, Ulsan, and Sejong metropolises

^b^Calculated as the total production of garlic × 73.6%

^c^Calculated as the total production of wet garlic stalk × 13.6%/86.4%

### Chemical composition

The chemical compositions of garlic stalk and rice straw are shown in Table 3. Garlic stalk had a higher (*P* < 0.05) content of DM, CP, ADF, ash, and NFC, and lower NDF and lignin contents than rice straw. The ADICP concentration did not differ between garlic stalk and rice straw.

**Table 3:** Chemical composition of garlic stalk and rice straw

Item, % of DM	Garlic stalk	Rice straw	SE	*P*-value
Dry matter, %	84.6	70.0	2.71	<0.001
Crude protein (CP)	5.5	4.0	0.53	0.018
Acid detergent insoluble CP, % of CP	35.3	29.6	4.52	0.385
Ether extract	1.5	0.9	0.15	0.006
Neutral detergent fiber (NDF)	54.9	70.6	1.48	<0.001
Acid detergent fiber	54.6	44.3	1.38	<0.001
Hemicellulose	0.3	26.3	2.40	<0.001
Ash	13.8	11.9	2.01	<0.001
Lignin	3.8	5.1	0.12	<0.001
Non-fiber carbohydrates^a^	24.3	12.7	2.09	<0.001

^a^Calculated as 100 − [CP + NDF + ether extract + ash]

### Ruminal degradation kinetics of DM and NDF

The in situ DM and NDF fractions of garlic stalk are shown in Table 4. The mean soluble A fraction of DM tended to be slightly greater (*P* = 0.06) in garlic stalk than in rice straw. The degradable B fraction of DM was 2.7-times greater in garlic stalk than in rice straw, resulting in a lower (2.55-fold decrease; *P* < 0.001) percentage of ruminally undegradable DM in garlic stalk than in rice straw. The degradation rate of the degradable B fraction for DM, within 48 h of rumen incubation, tended to be greater (*P* = 0.07) in garlic stalk than in rice straw. The mean soluble A fraction of NDF did not differ between garlic stalk and rice straw. The degradable B and undegradable C fractions were 2.03-times higher and 2.18-times lower in garlic stalk than in rice straw, respectively. Garlic stalk exhibited a higher fractional rate of NDF degradation than rice straw. Based on this rate, the time required for half the B fraction of NDF to be degraded in the rumen was estimated to be 12 h for garlic stalk.

**Table 4:** In situ fractionation of dry matter and neutral detergent fiber in garlic stalk and rice straw

Item	Garlic stalk	Rice straw	SE	*P*-value
Dry matter fractions (%)				
53-μm filterable and soluble A fraction	13.6	11.6	0.8	0.059
Degradable B fraction	60.5	22.4	3.0	<0.001
Undegradable C fraction^a^	25.9	66.0	2.8	<0.001
*K*_d_B^b^ (% h^−1^)	8.2	5.0	1.5	0.072
Neutral detergent fiber fractions (%)				
53-μm filterable and soluble A fraction	2.0	1.4	1.0	0.631
Degradable B fraction	68.0	33.5	4.2	<0.001
Undegradable C fraction	30.0	65.1	4.1	<0.001
*K*_d_B (% h^−1^)	7.8	3.3	0.9	0.001

^a^ Calculated as 100 − (A + B)

^b^ *K*_d_B = degradation rate of the degradable B fraction

The effective DM and NDF degradability of garlic stalk and rice straw are shown in Table 5. Effective degradability of DM, assuming a passage rate of 0.05 h^−1^, was found to be 2.3-fold greater in garlic stalk than in rice straw. Similarly, effective degradability of NDF, assuming a passage rate of 0.05 h^−1^, was 2.97-times greater in garlic stalk than in rice straw.

**Table 5:** In situ effective degradability of dry matter and neutral detergent fiber at two rates of rumen passage^a^

Item, %	Garlic stalk	Rice straw	SE	*P*-value
Effective degradability of DM				
*K*_p_B = 0.025, h^−1^	59.6	25.8	3.2	<0.001
*K*_p_B = 0.050, h^−1^	50.9	22.2	3.1	<0.001
Effective degradability of NDF				
*K*_p_B = 0.025, h^−1^	53.1	20.1	3.5	<0.001
*K*_p_B = 0.050, h^−1^	43.1	14.5	3.3	<0.001

^a^Effective degradability was calculated as: A + [B(*K*_d_B/(*K*_d_B + *k*p))], where A = soluble fraction, B = degradable fraction, *K*_d_B = degradation rate of the degradable B fraction, and *K*_p_ = the fractional rate of passage from the rumen

**Fig. 1:**
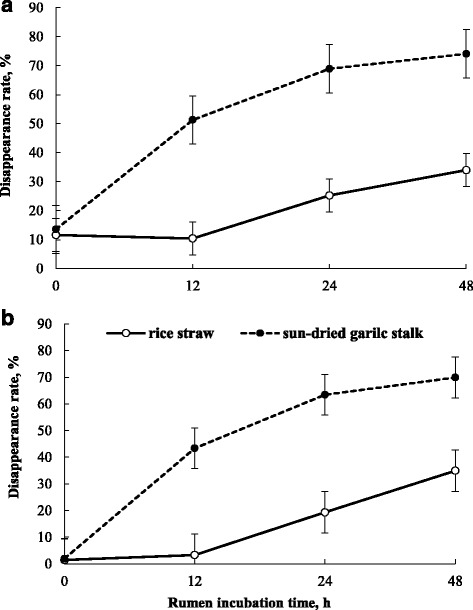
In situ dry matter (**a**) and neutral detergent fiber (**b**) disappearance of sun-dried garlic stalk and rice straw as a function of incubation in the rumen. Error bars indicate standard deviation

The rates of in situ DM and NDF disappearance from rice straw and garlic stalk, as a function of residence time (48 h) in the rumen, are illustrated in Fig. 1. The extent of DM and NDF disappearance from garlic stalk was considerably greater than that from rice straw; approximately 53.1 and 43.4% of DM and NDF in garlic stalk were disappeared during the first 12 h of incubation, compared to only 10.4 and 3.3% in rice straw, respectively.

### Total digestible nutrients of garlic stalk

The apparent digestibility coefficients of DM, EE, CP, CF, and NFE in the control diet (60% concentrate mix +40% ryegrass) were 63.6, 84.7, 62.0, and 72.6%, respectively, resulting in a TDN value of 63.8% (Table not presented). Likewise, total-tract digested DM, EE, CP, CF, and NEF in the treatment diet (70% control diet and 30% sun-dried garlic stalk) were 62.6, 68.6, 54.9, and 71.6%, respectively. The digestibility coefficients of DM, EE, CP, CF, and NFE in garlic stalk, calculated as the difference between the digestibility coefficients of control and treatment diets, were estimated to be 60.2, 20.4, 26.7, 72.6, and 68.0%, respectively, resulting in a TDN value of 56.3%.

## Discussion

The quantity of garlic stalk produced in 2013 was the highest of all the years included in this study, in the order of Jeonnam > Gyeongnam > Gyeongbuk > Jeju > Chungnam provinces, indicating that the highest quantities were produced in the southern regions. Garlic is the major overwintering crop widely cultivated in the southern and central regions of Korea, and is largely divided into tropical and temperate garlic [21]. Tropical garlic contributes to 77% of garlic production in Korea and its major production areas are high-temperature regions such as the southern coast, Jeju island, Shinhan, Muahn, Haenam, Goheung, and Namhae counties [22].

### Chemical composition

Although the mean values for CP and EE observed in the present investigation were lower than those reported by Chu et al. [9] for garlic stalk silage, comparable values were found for CF, ash, and NFE contents. Contents of CP and NDF, as reported by Kamruzzaman et al. [10] for garlic stem and leaf silage, were higher and lower than those obtained in our study, respectively. The chemical composition of garlic stalk showed that the NDF content was comparable to that of alfalfa [23]; however, the NDF content was lower than that found for timothy (83.4%) and ryegrass (78.9%) [24], which are imported in large amounts to Korea [25].

### In situ degradation kinetics

To our knowledge, no previous study has investigated the in situ DM and NDF degradation kinetics of garlic stalk; therefore, it was not possible to compare these values with those reported previously. Comparison of the in situ DM fractionations of garlic stalk in the present investigation with those reported for alfalfa hay by our research team [24] showed that garlic stalk exhibited a much lesser soluble A faction and greater degradable B fraction (60.5 vs. 21.8%) than alfalfa hay. The undegradable C fraction was also found to differ largely between the two feeds (25.9 vs. 46.3% for garlic stalk and alfalfa hay, respectively). A positive correlation exists between the soluble A fraction and the NFC content of feeds [26], which may help to explain the greater soluble A fraction of DM in garlic stalk compared with rice straw, as the difference in NFC content among the two feeds was large (24.3 vs. 12.7% for garlic stalk and rice straw, respectively).

The extent of NDF disappearance for garlic stalk was substantially greater than that for rice straw during a 48-h incubation in the rumen. Hoover [27] studied the factors involved in fiber digestion in the rumen, and found a negative correlation between lignin concentration and fiber digestion. Bruno-Soares et al. [28] also studied the NDF degradation kinetics of several legume straws and found that the concentration and composition of the cell walls (i.e., NDF and lignin) could best explain the variation in the potential degradability of NDF and DM among these straws. For example, these authors reported that the potential degradability of NDF was negatively correlated with NDF (*r* = −0.829) and lignin (*r* = −0.917). In the present study, garlic stalk was found to possess a lower concentration of lignin and NDF than rice straw, which may help to explain the difference in the rate of NDF disappearance for garlic stalk and rice straw. However, fiber digestion in the rumen is a very complex process and cannot be explained by the degree of lignification alone. Other factors, including the physical properties of cell walls, such as crystallinity and the degree of polymerization, are involved in the fiber digestion process [29].

The TDN value of garlic stalk (56.3%) was much higher than that of rice straw (44%), and comparable with that of medium-quality timothy hay (55.3%), as reported in the Standard Tables of Feed Composition in Korea [30].

## Conclusions

This study provides basic information on the kinetics of the ruminal DM and NDF degradation of garlic stalk, which are important for its efficient utilization in ruminant diet. The extent and rate of DM and NDF degradation in the rumen and thus TDN content were greater in garlic stalk than in rice straw, which appears to be a good source of roughage for ruminants, especially in places or countries where garlic stalk is abundantly generated. The longer-duration in vivo study with the varying inclusion levels of garlic stalk is recommended to evaluate the productive performance responses and determine the proper inclusion level in the diet.

## Abbreviations

ADF: Acid-detergent fiber
ADICP: Acid detergent insoluble CP
CF: Crude fiber
CP: Crude protein
DM: Dry matter
EE: Ether extract
NDF: Neutral-detergent fiber
NFC: Non-fiber carbohydrates
NFE: Nitrogen free extract
NPN: Non-protein nitrogen
TDN: Total digestible nutrients
